# The Impact of Sleep Timing, Sleep Duration, and Sleep Quality on Depressive Symptoms and Suicidal Ideation amongst Japanese Freshmen: The EQUSITE Study

**DOI:** 10.1155/2016/8737654

**Published:** 2016-03-03

**Authors:** Atin Supartini, Takanori Honda, Nadzirah A. Basri, Yuka Haeuchi, Sanmei Chen, Atsushi Ichimiya, Shuzo Kumagai

**Affiliations:** ^1^Department of Behavioral and Health Sciences, Graduate School of Human-Environment Studies, Kyushu University, 6-1 Kasuga-kouen, Kasuga, Fukuoka Prefecture 816-8580, Japan; ^2^Department of Environmental Medicine, Graduate School of Medical Science, Kyushu University, Maidashi 3-1-1, Higashi-ku, Fukuoka City, Fukuoka Prefecture 812-8582, Japan; ^3^Research Fellow of the Japan Society for the Promotion of Science, 5-3-1 Kojimachi, Chiyoda-ku, Tokyo 102-0083, Japan; ^4^Faculty of Arts and Science, Kyushu University, 6-1 Kasuga-kouen, Kasuga, Fukuoka Prefecture 816-8580, Japan

## Abstract

*Aim*. The aim of this study was to identify the impact of bedtime, wake time, sleep duration, sleep-onset latency, and sleep quality on depressive symptoms and suicidal ideation amongst Japanese freshmen.* Methods*. This cross-sectional data was derived from the baseline survey of the Enhancement of Q-University Students Intelligence (EQUSITE) study conducted from May to June, 2010. A total of 2,631 participants were recruited and completed the following self-reported questionnaires: the Pittsburgh Sleep Quality Index (PSQI), the Center for Epidemiologic Studies Depression Scale (CES-D), and the original Health Support Questionnaires developed by the EQUSITE study research team.* Results*. Of 1,992 participants eligible for analysis, 25.5% (*n* = 507) reported depressive symptoms (CES-D total score ≥ 16), and 5.8% (*n* = 115) reported suicidal ideation. The present study showed that late bedtime (later than 01:30), sleep-onset latency (≥30 minutes), and poor sleep quality showed a marginally significant association with depressive symptoms. Poor sleep quality was seen to predict suicidal ideation even after adjusting for depressive symptoms.* Conclusion*. The current study has important implications for the role of bedtime in the prevention of depressive symptoms. Improving sleep quality may prevent the development of depressive symptoms and reduce the likelihood of suicidal ideation.

## 1. Introduction

Depression is a disabling condition and a major public health issue worldwide [1]. The transition from high school to university life makes freshmen vulnerable to depression. Indeed, the prevalence of depression among university students is higher [2] than that in the general population, and it is increasing annually in Western countries [3]. Previous studies have indicated that depression among university students is a risk factor for poor academic performance [4], frequent illness [5], dropping out [6], problem drinking [7], and higher risk of suicidal ideation [8].

People aged 18 to 30 years report a higher prevalence of suicidal ideation and are more likely to have a suicide plan compared with older adults [9]. For young adults attending university, the risk of suicidal ideation is greater than that of their counterparts of the same age not attending university [10]. Japan has the highest global ranking for longevity; unfortunately, it also has one of the world's highest rates of suicide [11]. Indeed, suicide is the leading cause of death, followed by traffic accidents, among 15- to 19-year-olds in Japan [12]. A high proportion of students who commit suicide are clinically depressed; therefore, there is an urgent need to establish effective strategies to reduce the development of depression, which may lead to suicidal ideation among young adults in Japan.

Epidemiological studies have identified sleep disturbances as a significant risk factor for the later development of depression in healthy young adults [13]. Kaneita et al. [14] reported positive associations among insomnia, sleep duration, and poor mental health among adolescents. In another study using polysomnography, prolonged sleep latency [15] and short and long sleep duration were associated with an increased risk of depression [16, 17].

A neurobiological study suggested a correlation between circadian rhythm, a 24 h day-to-night cycle, and depression [18]. Chronotype, which refers to the timing of sleep and regular activities, has been suggested to affect circadian rhythm. In other clinical studies, the evening-type chronotype (late bedtime) was associated with a greater risk of depressive symptoms [19] and of suicide [20]. Given the lack of epidemiological studies on the impact of sleep timing on depressive symptoms and suicidal ideation among freshmen in Japanese universities, we performed the present study. Identifying the impact of sleep timing would facilitate establishment of effective strategies for reducing the development of depression and suicidal ideation.

In addition, there are some limitations of previous studies to be highlighted. First, most of sleep studies were performed in Western countries, and few have been conducted in Asia. Due to differences in lifestyle and culture, findings obtained in Western countries might not be applicable to Asian populations, Japan in particular. Secondly, whilst sleep quality contains quantity and quality parameters, the research on sleep and depression is mostly focusing only on the quantity of sleep (sleep duration). The study on sleep quality in relation with depression and suicide ideation is scarce.

Therefore, the main objective of the current study was to evaluate the impacts of sleep timing, sleep duration, sleep-onset latency, and sleep quality on depressive symptoms and suicidal ideation among Japanese freshmen. We hypothesised that late bedtime, late wake time, short sleep duration, prolonged sleep-onset latency, and poor sleep quality would have significant impacts on depressive symptoms and suicidal ideation.

## 2. Methods

### 2.1. Participants and Procedures

This was a baseline epidemiologic study on mental health improvement, known as the Enhancement for Q-University Students InTElligence (EQUSITE) study, among students at Kyushu University, a public university in southern Japan. The baseline study was conducted from May to June 2010.

**Figure 1:**
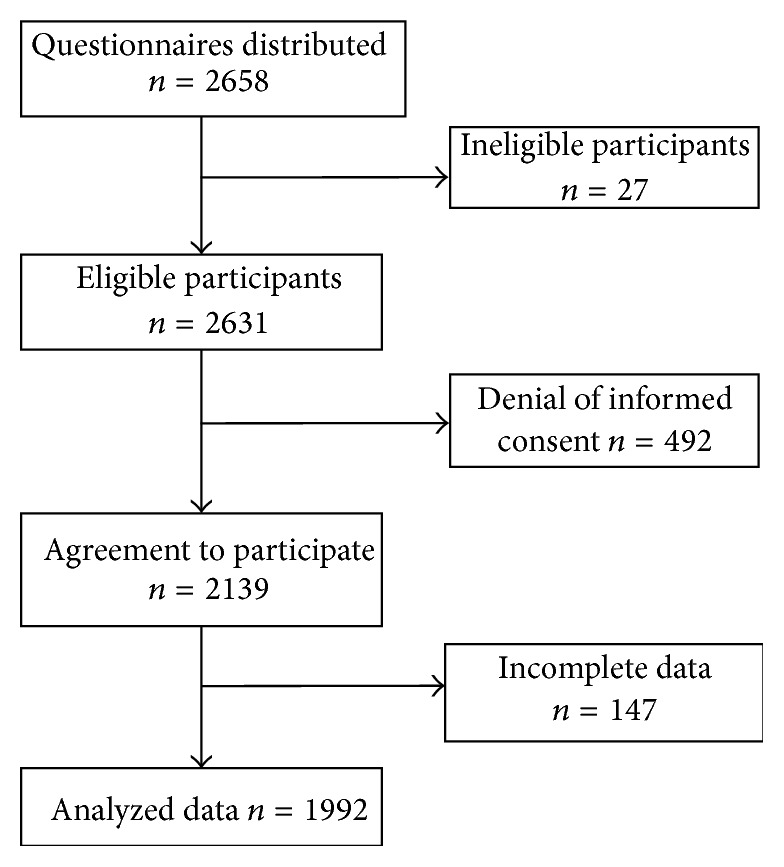
Flow chart of recruitment procedures.

The questionnaires were distributed to 2,658 students during physical education (PE), a compulsory class for freshmen. Of these students, 2,631 participants were freshmen and were thus eligible to participate in this study. Dates of birth, heights, and weights were collected during a routine health examination performed as a part of their orientation procedure. After excluding 639 participants due to lack of informed consent and missing data on sex, sleep, depression, and suicidal ideation, a total of 1,992 participants were included in the present analysis (Figure 1).

### 2.2. Measures

#### 2.2.1. Sociodemographic Characteristics and Health Behaviour

The Health Support Questionnaires developed by the EQUSITE study research team comprise questions regarding sociodemographic characteristics, lifestyle habits, and health conditions. Sociodemographic characteristics included in the analysis were age, sex, living condition (living alone in an apartment/living alone in a dormitory/living with parents), commute time to university (less than 30 min/more than 30 min), and yes or no responses to questions regarding part-time jobs and financial difficulty. The lifestyle variables included in this study were assessed on a binominal scale and concerned the following: alcohol consumption (never/once or more per week), smoking (never/sometimes), exercise habits (regular/irregular), exercise duration (less than 1 hour/more than 1 hour per week), and breakfast habits (regular/irregular).

Weight and height data were obtained from student's annual health examinations. Specialized health-examination nurses measured height and weight using standard protocols. Body mass index (BMI) was calculated as the weight in kilograms divided by the square of the height in meters. According to the standard BMI cut-offs of the World Health Organization, participants were classified as normal (BMI < 25) or overweight (BMI ≥ 25). Data on date of birth, height, and weight were collected during a routine health examination performed as part of the orientation procedure.

#### 2.2.2. Depressive Symptoms

The Japanese version of the Center for Epidemiologic Studies Depression Scale (CES-D) [21] was used to measure depressive symptoms. The CES-D is a self-report depression scale which has been widely used for identifying people with depressive symptoms in general population and has satisfactory levels of reliability and validity [22]. The CES-D comprises 20 items, each of which is scored from 0 to 3. The total score ranges from 0 to 60, with higher scores indicating increasing severity of depression. The Japanese version of CES-D has been translated into Japanese, and its reliability and validity have been confirmed in the Japanese general population [21]. Depressive symptoms were defined as a dichotomised variable with a universal cut-off score of 16 or higher [21, 22].

For the purpose of this study, sleep-related questions were excluded from the score to prevent their effects on associations between depression symptoms and sleep. The total CES-D score was calculated using the following formula: CES-D score = (sum of 19 item scores) × (20/19) × (19/number of answered questions). This method was used when five or fewer responses on the CES-D were missing [23–25].

#### 2.2.3. Suicidal Ideation

A physical and mental health-related questionnaire which contains a single suicidal ideation item was used for suicidal ideation assessment. Participants were asked the following questions: “Have you ever thought that you would be better off dead?” with response options of “yes” and “no.”

#### 2.2.4. Sleep Measurements

Bedtime, wake time, sleep duration, and insomnia were measured using the Japanese version of the Pittsburgh Sleep Quality Index (PSQI-J) which has been validated in Japanese population [26]. The PSQI is a self-administered questionnaire used to evaluate subjective sleep quality during the previous month [27]. It contains 19 items, each of which is scored from 0 to 3.

For the purpose of the current study, bedtime, wake time, and sleep duration were categorised. Epidemiology study on sleep timing is not yet well established. The study results of Horne and Östberg [28], however, indicated that a bedtime of 23:30 may be used as an indicator of a morning type among students or an evening type among adults [29]. Furthermore, Baehr et al. [30] stated that the body temperature minimum occurred at ~4 a.m. for the morning type and at 6 a.m. for the evening type. Therefore, for the sake of study analysis, we used the starting points of ≤23:30 for early bedtime and ≤6:00 for early wake time. Moreover, we categorised sleep duration into four categories: <6 h; 6-7 h; 7-8 h; and >8 h.

As for difficulty initiating sleep, we used the cut-off point of sleep-onset latency of more than 30 min [31]. Poor sleep quality was defined as a global PSQI score of >5.5 points [22].

#### 2.2.5. Ethical Considerations

Permission to conduct the study was obtained from the Ethics Committee of the Institute of Health Science, Kyushu University. Each questionnaire was accompanied by an informed consent form. Participants agreed to allow their questionnaire data and related examination results to be analysed, and all identifying information was kept confidential.

### 2.3. Statistical Analyses

All statistical analyses were performed using SAS version 9.3 (SAS Institute Inc., Cary, NC, USA). The chi-square test was used to compare subjects with and without depressive symptoms. Continuous variables were compared using independent-sample *t*-tests. For all comparisons, the statistical level of significance was set at *p* < 0.05.

We evaluated the effects of sleep timing, sleep duration, and insomnia symptoms on the risk of developing depressive symptoms and suicidal ideation in separate multivariate-adjusted models. Multiple factorial logistic regression was performed to estimate the odds ratio (OR) and 95% confidence interval (CI) of depressive symptoms for each category of bedtime, wake time, sleep duration, sleep-onset latency, and sleep quality using a bedtime earlier than 23:30 p.m., a wake time earlier than 6:00 a.m., or 7 h to 8 h sleep duration as the reference.

The first multiple logistic regression model (model 1) was adjusted for age and sex. In the second model (model 2), we further adjusted for age, sex, exercise habits, exercise duration, breakfast habits, drinking habits, smoking habits, BMI, financial difficulty, commute time to campus, and part-time job. In the other model (model 3), we added sleep duration that may confound the relationship between the risk factors and depressive symptoms, which was treated as an outcome. In the evaluation of suicidal ideation and sleep behaviours, we did not add sleep duration to model 3; however, we further added depressive symptoms as a covariate.

In addition, we performed sensitivity analyses. Analysis of those with incomplete data yielded results similar to the main analysis (data not shown). In addition, the results for sleep duration were unaffected by the use of other categorisations for sleep duration (data not shown).

## 3. Results

### 3.1. Sociodemographic Characteristics of Participants

The characteristics of participants according to depressive symptoms are presented in Table 1. The average age of students was 18.4 ± 1.1 years. Of the 1992 participants, almost one-quarter (*n* = 521, 25.4%) were identified as having depressive symptoms, and 5.77% (*n* = 115) reported suicidal ideation. Freshmen with depressive symptoms had a markedly higher risk of suicidal ideation (*n* = 82, 4.12%).

In the bivariate analysis, no statistically significant association between depressive symptoms and sex was found (*p* = 0.4).

### 3.2. Associations between Depressive Symptoms and Sleep Behaviours

Table 2 presents the results of our bivariate analysis of sleep behaviours according to depressive symptoms and suicidal ideation. Depressive symptoms were significantly associated with bedtime (*p* = 0.01), sleep-onset latency (*p* < 0.001), and poor sleep quality (*p* < 0.001). However, no statistically significant association was found between depressive symptoms and either wake time or sleep duration.

Multiple logistic regression analyses were conducted to further examine the association between depressive symptoms and bedtime, wake time, sleep-onset latency, sleep duration, and sleep quality. As detailed in Table 3, late bedtime was significantly associated with an increased prevalence of depressive symptoms even after adjustment for potential confounders; the multivariate-adjusted OR of depression for bedtime of later than 01:30 versus 23:30 or earlier was 1.59 (95% CI: 1.04–2.44). The multivariate-adjusted OR of depressive symptoms was attenuated after adjustment for sleep duration, and the association was not significant (OR: 1.56, 95% CI: 0.99–2.47).

Sleep-onset latency of more than 30 minutes was significantly associated with an increased prevalence of depressive symptoms (OR = 1.53; 95% CI: 1.22–1.93). This association remained significant after adjustment for sleep duration (OR = 1.52; 95% CI: 1.21–1.92).

Poor sleep quality was significantly associated with an increased prevalence of depressive symptoms in age and sex adjusted model (OR = 2.49; 95% CI: 2.02–3.06). The multivariate-adjusted OR for depressive symptoms and poor sleep quality remained significant after adjustment for all covariates (OR = 2.45; 95% CI: 1.98–3.04) and sleep duration (OR = 2.71; 95% CI: 2.14–3.45).

Similar to the bivariate analysis, the multivariate-adjusted model revealed no significant associations between sleep duration and depressive symptoms.

### 3.3. Associations between Suicidal Ideation and Sleep Behaviours

**Figure 2:**
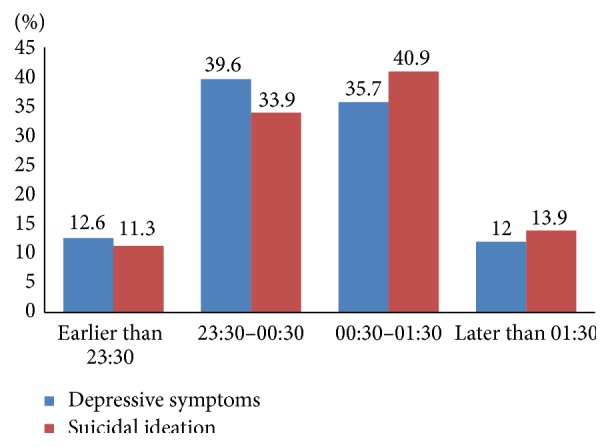
Prevalence of depressive symptoms and suicidal ideation according to bedtime.

Figure 2 showed that the prevalence of depressive symptoms and suicidal ideation was higher as the sleep timing delayed. However, the results of multiple logistic regression analyses for suicidal ideation presented in Table 4 indicated that bedtime was not significantly associated with suicidal ideation. Sleep duration and sleep-onset latency were also not significantly associated with suicidal ideation.

Poor sleep quality, however, was significantly associated with an increased prevalence of suicidal ideation (OR = 2.44; 95% CI: 1.65–3.59). After adjustment for depressive symptoms, the association remained significant (OR = 1.71; 95% CI: 1.13–2.57).

## 4. Discussion

The aim of this study was to examine the effects of sleep behaviours (i.e., bedtime, wake time, sleep-onset latency, sleep duration, and sleep quality) on depressive symptoms and suicidal ideation in university freshmen in Japan. To our knowledge, previous studies have not addressed the association between bedtimes, wake time, sleep-onset latency, sleep duration, and sleep quality on depressive symptoms and suicidal ideation among university freshmen in Japan using a large sample. Thus, this study may facilitate the development of new behavioural treatment strategies for reducing the development of depressive symptoms and suicidal ideation in freshmen.

Our results suggested that the prevalence of depressive symptoms was significantly higher among freshmen with a late bedtime, prolonged sleep-onset latency, and poor sleep quality. However, bedtime and prolonged sleep-onset latency showed no significant association with suicidal ideation. As for wake time, it showed a significant association with neither depressive symptoms nor suicidal ideation. In contrast to previous findings, we could not find a significant association either between sleep duration and depressive symptoms or suicidal ideation. In addition, our findings showed a significant association between sleep quality and suicidal ideation, even after adjusting for depressive symptoms.

### 4.1. Associations among Bedtime, Depressive Symptoms, and Suicidal Ideation

Previous studies have reported a significant association between evening chronotype and depressive symptoms [19, 32]. A study of almost 16,000 adolescents found an association between earlier parental set bedtime and depression as well as suicidal ideation [33]. Recently, a study on depressive symptoms among young adults of similar age as in this study in Japan found that late bedtime and short sleep duration were associated with an increased risk of depressive symptoms [34]. Consistent with these findings, our multiple logistic regression analyses found that a late bedtime (later than 01:30) was significantly associated with an increased likelihood of being depressed. However, the association between late bedtime and depressive symptoms was attenuated after adjustment for sleep duration. Similarly, Sakamoto et al. [25] could not find the association between bedtime and depressive symptoms in Japanese workers after adjustment for sleep duration. In this regard, as bedtime was closely associated with sleep duration, he has suggested the analysis without adjustment for sleep duration to provide a better estimate of the true association between late bedtime and depressive symptoms than analysis with such adjustment.

The mechanisms underlying the association between bedtime and depression remain unknown. However, chronobiological studies suggest the existence of functional associations among sleep timing preference, the regulatory function of the biological clock, and mood adjustments [35]. Baehr et al. noted that later sleep timing increases the risk of depressive symptoms because of the internal desynchronisation between sleep timing preferences and physiological rhythms [30]. In the present study, the association between bedtime and suicidal ideation could not be detected.

### 4.2. Associations among Sleep Duration, Depressive Symptoms, and Suicidal Ideation

Previous studies have reported that sleep duration is associated with an increased risk of depression [16, 23]. A recent meta-analysis on sleep duration and depression has also indicated that short and long sleep duration are significantly associated with increased risk of depression in adults [36]. Short sleep duration has also been associated with an increased risk for suicidal ideation in a cross-sectional study of Korean adults [37]. In contrast, our study did not show a significant association between sleep duration and depression or suicidal ideation. These findings have several possible explanations. First, the definitions of “short” and “long” sleep durations are inconsistent among studies. Second, previous studies have used adjustments for various covariates, such as sociodemographic factors, health-related behaviour, and medication, that may have influenced the results. Last but not least, the size of sample population may affect the significant association between sleep duration and depressive symptoms or suicidal ideation.

### 4.3. Associations among Sleep Quality, Depressive Symptoms, and Suicidal Ideation

To date, there are few epidemiological studies that addressed the association between sleep quality, depressive symptoms, and suicidal ideation. Previous study, mostly, associated depressive symptoms and suicidal ideation with sleep problem such as insomnia [13, 38, 39]. A longitudinal study in elderly indicated that poor subjective sleep quality is associated with increased risk for death by suicide, even after adjustment for depressive symptoms. In a clinical study, sleep quality revealed as a significant determinant of onset of major depression [20]. In line with these studies, our multiple logistic regression analyses found that sleep quality was significantly associated with depressive symptoms and suicidal ideation [40]. The association between sleep quality and suicidal ideation remained significant even after adjustment for depressive symptoms. Poor sleep quality was shown to have a higher likelihood of depressive symptoms and suicidal ideation compared to those who have a good sleep quality. This demonstrated the importance of targeting sleep quality in the young age to prevent the development of depressive symptoms and suicidal ideation in the later life.

Suicidal ideation has been linked to psychological mechanism such as hopelessness [41] and pessimism [42]. Poor sleep quality leads to depressive mood, which in turn induces pessimism [42]. Fortier-Brochu et al. [43] describe that insomnia is associated with deficient problem-solving capacity, especially when involving complex tasks. Thus, insomnia leads to perceived deficiencies in decision-making that may lead depressed individual to consider a suicide as a solution. We recognize that insomnia and poor sleep quality are different problems. However, insomnia may affect the quality of sleep.

### 4.4. Associations among Sleep-Onset Latency, Depressive Symptoms, and Suicidal Ideation

A study using polysomnography indicates that prolonged sleep-onset latency is associated with an increased risk of depression [15]. In contradiction, a study in middle-aged adults conducted in Japan showed that difficulty maintaining sleep predicts death by suicide but not with difficulty initiating sleep [44]. Furthermore, Sabo et al. [45] indicate that suicide attempters have longer sleep latency. In the present study, our data supported the association between prolonged sleep-onset latency and depressive symptoms. However, the association between prolonged sleep-onset latency with suicidal ideation could not be found.

Even though the association between sleep-onset latency and suicidal ideation was not detected, sleep-onset latency and depressive symptoms were significantly associated. As depression carries a high risk of suicide, therefore, sleep-onset latency might have indirect relationship through depressive symptoms. Further research is needed to identify the direct-indirect relationship among sleep-onset latency, depressive symptoms, and suicidal ideation.

### 4.5. Strengths and Limitations

To our knowledge, this is one of the few epidemiological studies to include a relatively high rate of participation by college freshmen. Second, our study highlighted bedtime and wake time as potential risk factors for depressive symptoms. In addition, we adjusted for other sleep variables in each model to analyse the relationship between each sleep variable and depressive symptoms as well as suicidal ideation.

Several limitations should be considered when interpreting the present results. First, as this was a cross-sectional study, we could not rule out reverse causality. A longitudinal study is, therefore, required to confirm the results. Second, objective diagnostic assessments of sleep and depression were not used in the present study; the use of such measures would increase the accuracy of the data. However, self-reported questionnaires appear to be more effective for collecting data in epidemiologic studies involving a large number of participants. We used the CES-D, which is a commonly used nondiagnostic tool for depressive symptoms. The reliability and validity of the CES-D for epidemiologic studies are widely recognised [22, 29, 46]. Additionally, the PSQI has been used to assess sleep quality worldwide and has been validated in Japan [26]. Last but not least, suicidal ideation has been assessed by a single-item which may lead to misclassification. Improving the measurement of suicidal ideation assessment is critical to increase the accuracy of the result.

## 5. Conclusions

In conclusion, the present study revealed a high frequency of depressive symptoms amongst university freshmen in southern Japan. As hypothesised, depressive symptoms were closely associated with later bedtime, prolonged sleep-onset latency, and poor sleep quality. Furthermore, suicidal ideation was significantly associated with poor sleep quality only. Our study demonstrated that poor sleep quality impacted suicidal ideation independent of depressive symptoms.

The mechanisms of sleep, depressive symptoms, and suicidal ideation have been linked to biological and psychological mechanisms. Understanding these mechanisms, we may suggest that sleep timing, sleep-onset latency, and sleep quality can be an independent risk factor for depressive symptoms.

The current study has important implications for the role of bedtime in the prevention of depressive symptoms. Improving sleep quality may prevent the development of depressive symptoms and reduce the likelihood of suicidal ideation.

## Figures and Tables

**Table 1:** The characteristics of study participants with and without the depressive symptoms.

	Total	Nondepressed (*n* = 1485)	Depressed (*n* = 507)	*p* value
	*n* (%)	*n* (%)	*n* (%)	
Age, years (means ± standard deviations)	18.4 ± 1.1	18.4 ± 1.1	18.4 ± 1.1	0.28
Gender				0.40
Male	1385 (69.5)	1026 (69.1)	359 (70.8)	
Female	607 (30.5)	459 (30.9)	148 (29.2)	
Weight category				0.36
Normal weight (BMI < 25 kg/m^2^)	1786 (89.7)	1326 (89.3)	460 (90.7)	
Overweight (BMI ≥ 25 kg/m^2^)	206 (10.3)	159 (10.7)	47 (9.3)	
Exercise habits				<0.01
Irregular	724 (36.3)	494 (33.3)	230 (45.4)	
Regular	1268 (63.7)	991 (66.7)	277 (54.6)	
Exercise duration				
<1 h	1109 (55.7)	784 (52.8)	325 (64.1)	<0.01
≥1 h	883 (44.3)	701 (47.2)	182 (35.9)	
Breakfast habits				<0.01
Irregular	466 (23.4)	315 (21.2)	151 (29.8)	
Regular	1526 (76.6)	1170 (78.8)	356 (70.2)	
Smoking habits				0.05
Never	1969 (98.8)	1472 (99.1)	497 (98.0)	
Sometimes	23 (1.2)	13 (0.9)	10 (2.0)	
Drinking habits				0.98
Never	1229 (61.7)	916 (61.7)	313 (61.7)	
Once or more per week	763 (38.3)	569 (38.3)	194 (38.3)	
Financial difficulty				0.02
No	1761 (88.4)	1327 (89.4)	434 (85.6)	
Yes	231 (11.6)	158 (10.6)	73 (14.4)	
Living condition				
Living alone	1297 (65.1)	958 (64.5)	339 (66.9)	0.13
Living in dormitory	136 (6.8)	95 (6.4)	41 (8.1)	
Living with parents	559 (28.1)	432 (29.1)	127 (25.0)	
Commute time				
<30 min	893 (44.8)	648 (43.6)	245 (48.3)	0.07
≥30 min	1099 (55.2)	837 (56.4)	262 (51.7)	
Part-time job				0.06
No	1593 (80.0)	1173 (79.0)	420 (82.8)	
Yes	399 (20.0)	312 (21.0)	87 (4.4)	
Suicidal ideation				
No	1877 (94.2)	1452 (97.8)	425 (21.3)	<0.01
Yes	115 (5.8)	33 (2.2)	82 (4.1)	

**Table 2:** Sleep behaviours according to depressive symptoms and suicidal ideation.

	Total	Nondepressed (*n* = 1485)	Depressed (*n* = 507)	*p* value	No suicidal ideation (*n* = 1877)	Suicidal ideation (*n* = 115)	*p* value
	*n* (%)	*n* (%)	*n* (%)		*n* (%)	*n* (%)	
Bedtime							
23:30 or earlier	287 (14.4)	223 (15.0)	64 (12.6)	0.01	274 (14.6)	13 (11.3)	0.04
23:30–00:30	860 (43.2)	659 (44.3)	201 (39.6)		821 (43.7)	39 (33.9)	
00:30–01:30	663 (33.3)	482 (32.5)	181 (35.7)		616 (32.8)	47 (40.9)	
Later than 01:30	182 (9.1)	121 (8.1)	61 (12.0)		166 (8.8)	16 (13.9)	
Wake time							
06:00 or earlier	935 (46.9)	718 (48.4)	217 (42.8)	0.15	882 (47.0)	53 (46.1)	0.65
06:00–07:00	762 (38.3)	555 (37.4)	207 (40.8)		717 (38.2)	45 (39.1)	
07:00–08:00	242 (12.2)	176 (11.9)	66 (13.0)		230 (12.3)	12 (10.4)	
Later than 08:00	53 (2.7)	36 (2.4)	17 (3.4)		48 (2.6)	5 (4.3)	
Sleep-onset latency							0.17
<30 min	1526 (76.6)	1170 (78.8)	356 (70.2)	<0.01	1444 (76.9)	82 (71.3)	
≥30 min	466 (23.4)	315 (21.2)	151 (29.8)		433 (23.1)	33 (28.7)	
Sleep duration							
6 h or less	551 (27.7)	421 (28.4)	130 (25.6)	0.34	510 (27.2)	41 (35.7)	0.23
6-7 h	932 (46.8)	699 (47.1)	233 (46.0)		886 (47.2)	46 (40.0)	
7-8 h	417 (20.9)	298 (20.1)	119 (23.5)		395 (21.0)	22 (19.1)	
>8 h	92 (4.6)	67 (4.5)	25 (4.9)		86 (4.6)	6 (5.2)	
Sleep quality				<0.01			<0.0001
Good sleep	1329 (66.7)	1070 (72.1)	259 (51.1)		1278 (68.1)	51 (44.3)	
Poor sleep	663 (33.3)	415 (28.0)	248 (48.9)		599 (32.0)	64 (55.7)	

**Table 3:** Association between sleep behaviours and depressive symptoms according to multinominal logistic regression analysis.

	Model 1	Model 2	Model 3
	OR (95% CI)	OR (95% CI)	OR (95% CI)
Bedtime			
23:30 or earlier	1	1	1
23:30–00:30	1.06 (0.77–1.46)	1.07 (0.78–1.49)	1.11 (0.79–1.55)
00:30–01:30	1.30 (0.94–1.81)	1.32 (0.95–1.84)	1.32 (0.92–1.89)
Later than 01:30	**1.75 (1.15–2.65)**	** 1.60 (1.05–2.44)**	1.57 (0.99–2.47)
Wake time			
06:00 or earlier (ref)	1	1	1
06:00–07:00	1.07 (0.83–1.38)	1.04 (0.80–1.35)	1.13 (0.86–1.47)
07:00–08:00	1.28 (0.95–1.72)	1.17 (0.85–1.63)	1.33 (0.95–1.85)
Later than 08:00	1.20 (0.72–1.98)	1.07 (0.63–1.76)	1.18 (0.68–2.05)
Sleep-onset latency			
<30 minutes (ref)	1	1	1
≥30 minutes	** 1.57 (1.25–1.98)**	** 1.53 (1.22–1.93)**	** 1.52 (1.21–1.92)**
Sleep duration			
6 h or less (ref)	1.21 (0.91–1.61)	1.31 (0.97–1.76)	
6-7 h	0.97 (0.74–1.28)	1.04 (0.78–1.37)	
7-8 h	1	1	
>8 h	1.36 (0.87–2.13)	1.36 (0.87–2.14)	
Sleep quality			
Good sleep (ref)	1	1	1
Poor sleep	** 2.49 (2.02–3.06)**	**2.45 (1.98–3.04)**	** 2.71 (2.14–3.45)**

OR: odds ratio; CI: confidence interval.

Model 1: adjusted for age, gender.

Model 2: adjusted for age, gender, weight category, exercise habits, exercise duration, breakfast habits, smoking habits, drinking habits, financial difficulty, living condition, commute time, and part-time job.

Model 3: adjusted for all variables in model 2 and sleep duration.

**Table 4:** Association between sleep behaviours and suicidal ideation according to multiple logistic regression analysis.

	Model 1	Model 2	Model 3
	OR 95% CI	OR 95% CI	OR 95% CI
Bedtime			
23:30 or earlier (ref)	1	1	1
23:30–00:30	1.02 (0.54–1.95)	1.01 (0.53–1.93)	1.03 (0.53–2.02)
00:30–01:30	1.64 (0.87–3.10)	1.66 (0.87–3.14)	1.56 (0.80–3.04)
Later than 01:30	2.04 (0.95–4.37)	1.88 (0.86–4.09)	1.53 (0.68–3.46)
Wake time			
06:00 or earlier (ref)	1	1	1
06:00–07:00	0.66 (0.42–1.02)	0.71 (0.44–1.13)	0.68 (0.42–1.12)
07:00–08:00	0.70 (0.40–1.22)	0.74 (0.40–1.34)	0.66 (0.35–1.25)
Later than 08:00	0.88 (0.36–2.16)	0.86 (0.34–2.17)	0.86 (0.33–2.26)
Sleep-onset latency			
<30 min (ref)	1	1	1
≥30 min	1.34 (0.88–2.04)	1.28 (0.84–1.96)	1.03 (0.66–1.61)
Sleep duration			
6 h or less (ref)	1.73 (0.99–3.03)	1.68 (0.94–2.99)	1.52 (0.83–2.79)
6-7 h	1.31 (0.75–2.29)	1.31 (0.74–2.31)	1.38 (0.77–2.50)
7-8 h	1	1	1
>8 h	0.62 (0.25–2.26)	0.74 (0.24–2.25)	0.61 (0.20–1.92)
Sleep quality			
Good sleep (ref)	1	1	1
Poor sleep	** 2.65 (1.81–3.89)**	** 2.44 (1.65–3.59)**	** 1.71 (1.13–2.57)**

OR: odds ratio; CI: confidence interval.

Model 1: adjusted for age, gender.

Model 2: adjusted for age, gender, weight category, exercise habits, exercise duration, breakfast habits, smoking habits, drinking habits, financial difficulty, living condition, commute time, and part-time job.

Model 3: adjusted for all variables in model 2 and depressive symptoms.
